# WRN helicase defective in the premature aging disorder Werner syndrome genetically interacts with topoisomerase 3 and restores the *top3* slow growth phenotype of *sgs1 top3*

**DOI:** 10.18632/aging.100020

**Published:** 2009-02-05

**Authors:** Monika Aggarwal, Robert M. Brosh

**Affiliations:** Laboratory of Molecular Gerontology, National Institute on Aging, NIH,; NIH Biomedical Research Center, Baltimore, MD 21224 USA

**Keywords:** Werner syndrome, helicase, topoisomerase, RecQ, Bloom's syndrome, Sgs1, genomic instability

## Abstract

Werner syndrome (WS) is a premature aging disorder characterized by genomic instability. The *WRN* gene defective in WS encodes a protein with both helicase and exonuclease activities that interacts with proteins implicated in DNA metabolism.  To understand its genetic functions, we examined the ability of human WRN to rescue phenotypes associated with *sgs1*, the sole RecQ helicase in *Saccharomyces cerevisiae*.  WRN failed to rescue *sgs1 *sensitivity to the DNA damaging agent methylmethane sulfonate or replication inhibitor hydroxyurea, suggesting divergent functions of human and yeast RecQ helicases.  However, physiological expression of WRN in *sgs1 top3* restored *top3 *slow growth phenotype, whereas no effect on growth was observed with wild-type or *sgs1 *strains.  Slow growth of WRN-transformed *sgs1 top3* correlated with an elevated population of large-budded cells with undivided nuclei, indicating restoration of cell cycle delay in late S/G2 characteristic of *top3*.  WRN helicase but not exonuclease activity was genetically required for restoration of *top3 *growth phenotype, demonstrating separation of function of WRN catalytic activities.  A naturally occurring missense polymorphism in WRN that interferes with helicase activity abolished its ability to restore *top3* slow growth phenotype.  Proposed roles of WRN in genetic pathways important for the suppression of genomic instability are discussed.

## Introduction

Understanding the genetic pathways of the WRN helicase-exonuclease has posed a complex challenge to researchers.  Studies of WRN-deficient cell lines have provided evidence for a role of WRN in the response to replicational stress through a recombinational repair pathway [1,2]; however, the precise molecular functions and protein interactions required for WRN to help cells proliferate, maintain genomic stability, and deal with endogenous or exogenously induced DNA damage are not well understood.  Moreover, although the clinical and cellular phenotypes of Werner Syndrome (WS)  appear to be distinct from that of  the other human RecQ helicase disease, it is not clear if WRN has entirely unique or at least partially overlapping roles with the other RecQ helicases to maintain genomic stability (for review, see [3,4]).

To investigate the genetic functions of WRN in a defined setting, we have developed a yeast-based model system to study the functional requirements of WRN in pathways that are conserved between yeast and human.  Unlike human cells which have five RecQ helicases (WRN, BLM, RECQ1, RECQ5, and RECQ4), *Saccharomyces cerevisiae* has only a single RecQ homolog, Sgs1 [4].  Although *sgs1* mutants exhibit sensitivity to DNA damaging agents or replication inhibitors and display a shortened lifespan, the best known genetic function of *sgs1* is to suppress the slow growth phenotype of a *top3* mutant.  It is believed that Top3 decatenates intertwined DNA molecules generated by Sgs1 helicase during replication [5,6]; therefore, in the absence of Top3, torsional stress is not relieved resulting in slow growth and hyper-recombination.  Based on the genetic and physical interaction of Sgs1 with Top3 [5,7,8], a model has been proposed that together they suppress the formation of cross-over products that arise from the resolution of Holliday Junction (HJ) recombination intermediates [9,10]. Conserved interactions between RecQ helicases and Top3 exist in other organisms as well [11-15].

In human cells, BLM physically interacts with Top3α, and  the two proteins together have the ability to catalyse double HJ dissolution on model DNA substrates in a reaction that requires BLM-mediated ATP hydrolysis and the active-site tyrosine residue of Top3α [16].  This reaction gave rise exclusively to non-cross-over products, as predicted from the hemicatenane model, and supports a proposed role of BLM with Top3α as a suppressor of sister chromatid exchanges (SCEs).  RMI1 (BLAP75) promotes this BLM-dependent dissolution of the homologous recombination (HR) intermediate by recruiting Top3α to the double HJ [17,18]. Interestingly, BLM appears to be unique in the double HJ dissolution reaction since WRN, RECQ1 and RECQ5 all failed to substitute for BLM [17,19]. Moreover, association of Top3α and BLAP75 with BLM stimulates its HJ unwinding activity; however, neither WRN nor *E. coli* RecQ HJ unwinding was stimulated by Top3α BLAP75 [20]. Very recently, a new component of the BLM-Top3α complex, designated RMI2, was identified that is important for the stability of the BLM protein complex [21,22].  RMI2 deficiency in vertebrate cells results in chromosomal instability [21,22], suggesting its function as a tumor suppressor. RMI2 enhanced the double HJ dissolvase activity of the BLM-Top3α complex [21], indicating that additional proteins are likely to be involved.  In fact, other proteins were isolated with the RMI2 complex, including the mismatch repair complex MSH2/6, RPA, and the Fanconi Anemia proteins FANCM and FAAP24 [21].

The suppression of recombinant cross-over products that are detected as sister chromatid exchanges is thought to be specific to the coordinate functions of yeast Sgs1 and Top3, and its human counterparts, BLM and Top3α However, RECQ5 and RECQ1 also interact with Top3α[23,24], and elevated SCE is also found in fibroblasts from RECQ5 [25] or RECQ1 [26] knockout mice as well as human cells depleted of RECQ1 by RNA interference [27].  These studies suggest that RecQ helicases participate in non-redundant pathways to suppress cross-overs during mitosis [28].

WS cells have a unique form of genomic instability known as variegated translocation mosaicism, characterized by extensive deletions and rearrangements [29].  WS cells, like other RecQ mutants, are also defective in recombination and sensitive to DNA damaging agents [3,30].  To begin to understand the basis for these defects in cellular DNA metabolism, we tested the ability of WRN in defined genetic backgrounds using yeast as a model system.  Using this approach, we discovered that WRN restores the *top3* slow growth phenotype in the *sgs1 top3* background, but does not complement the DNA damage sensitivity or growth of a single *sgs1* mutant.  WRN helicase activity is required for genetic restoration of the *top3* growth phenotype, suggesting that WRN unwinding activity creates a DNA substrate* in vivo* that in the absence of *top3 *prevents normal cell growth.  These results have implications for the potentially overlapping pathways between RecQ helicases, particularly WRN and the human homolog of Sgs1, BLM.  Moreover, depending on the genetic background, recombinational events initiated or mediated by WRN helicase activity may have consequences for cell cycle progression and cell growth.

## Results

### Expression of the human Werner syndrome protein (WRN) in yeast fails to rescue the methylmethanesulfonate or hydroxyurea hypersensitivity of an *sgs1 *mutant

**Figure 1.**
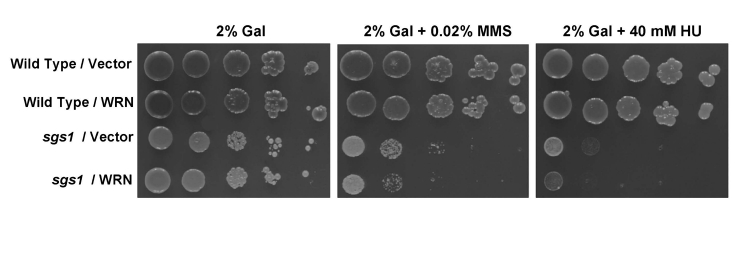
WRN fails to rescue the MMS and HU sensitivity of *sgs1*. Cultures of wild-type parental strain (W303-1A) or *sgs1* strain transformed with YEp112SpGAL or YEp112SpGAL-WRN were grown to early log phase (*OD *_600_ of ~0.6 to 0.8). Ten-fold serial dilutions of these cultures were spotted onto SC-Trp plates containing 2% gal and either MMS or HU at the indicated concentrations.  Plates were incubated at 30°C for 3 days (control plates) and 5 days (MMS or HU plates) and then photographed.

The *sgs1* strain is characterized by sensitivity to the compound methylmethanesulfonate (MMS), which introduces alkylation base damage or to hydroxyurea (HU), a replication inhibitor.  To assess the effect of WRN expression on the MMS or HU-sensitivity phenotype of the *sgs1* mutant, *sgs1* was transformed with a multi-copy TRP1 selectable plasmid which is either empty or encoding full-length WRN protein (1-1432).  Quantitative Western blot analyses using purified recombinant WRN protein as standard indicated that 8.1 x 104WRNmolecules/cell were present at 2% galactose (gal) concentration (data not shown).   In comparison, the level of endogenous WRN in HeLa cells was determined to be  8.9 x 104 molecules/cell [31], in agreement with published values for WRN copy number in other human cells [32,33]. Therefore, the level of WRN protein expression in yeast is comparable to that in human cells and considered physiological.  The *sgs1 *strain transformed  with empty vector  (*sgs1/ /*vector) did not expressprotein specifically recognized by WRN antibody (data not shown).  Transformed *sgs1* cells were serially diluted and spotted on to a synthetic complete (SC) 2% gal media lacking tryptophan (Trp) and containing the indicated concentration of either MMS or HU.  As shown in Figure 1, WRN failed to rescue the sensitivity of the *sgs1 *mutant to MMS or HU, and was observed to enhance the sensitivity of *sgs1* to either drug.  The vector-transformed wild-type parent strain (W3031A) was included as a control, showing resistance to MMS or HU (Figure 1).  The wild-type parent strain transformed with the YEp112SpGAL-WRN plasmid (Wild type/WRN) showed MMS and HU resistance comparable to Wild type/Vector (Figure 1), indicating that the drug sensitivity was dependent on the *sgs1* mutation.  Similar results were obtained at lower WRN protein levels induced by 0.5% gal (Supp. Data Figure 1).

### WRN and TOP3 genetically interact

Strains mutant for *top3* have pleiotropic phenotypes including severe growth defect, hyper-recombination at multiple loci, and sensitivity to the DNA damaging agents MMS and HU.  However, a mutation in the *sgs1* gene suppresses the slow growth rate of *top3 *[5].  Since it has been proposed that Top3 may be an evolutionarily conserved partner of RecQ helicases, we examined if the expression of  WRN could  substitute for Sgs1  and hence confer slow growth in the *sgs1top3* background.

**Figure 2.**
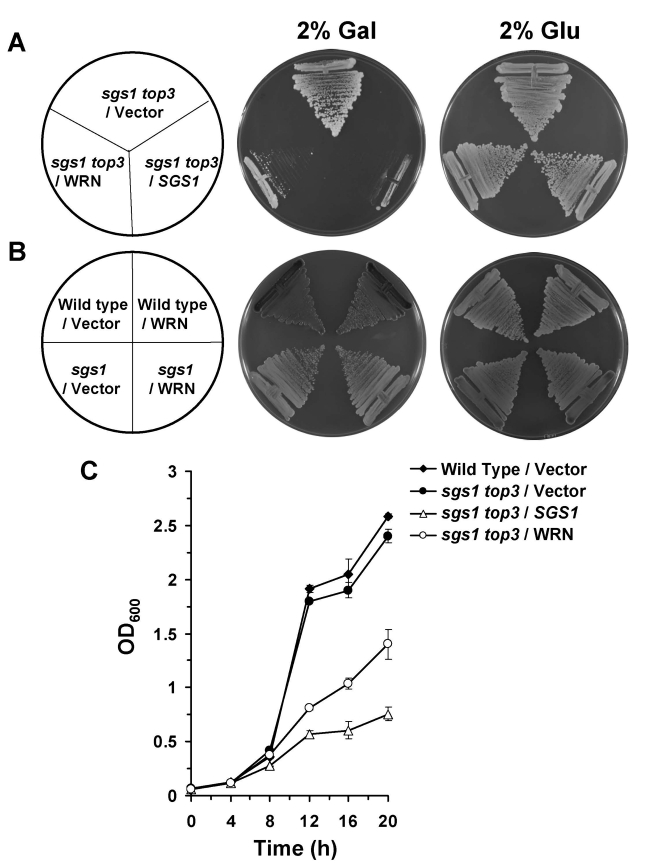
WRN expression in *sgs1 top3* restores the slow growth phenotype of *top3*. *Panel **A***, *sgs1 top3 *strain transformed with YEp112SpGAL or YEp112SpGAL-WRN were streaked on an SC-Trp plate containing either 2% glu or 2% gal.  As a control *sgs1 top3* strain transformed with YEp112SpGAL-*SGS1* was streaked on both the plates.  Plates were incubated at 30°C for 2 days and then photographed.  *Panel **B***, Wild type parental strain W303-1A or *sgs1* strain transformed with YEp112SpGAL or YEp112SpGAL-WRN were streaked on an SC-Trp plate either containing 2% glu or 2% gal.  Plates were incubated at 30°C for 4 days and then photographed.  Composition of the plates was as in *Panel **A*** and *Panel **B*** respectively.  *Panel **C***, Comparison of growth of *sgs1 top3 *strain transformed with YEp112SpGAL, YEp112SpGAL-WRN or YEp112SpGAL-*SGS1*.  Logarithmically growing cultures of above mentioned strains were reinoculated at OD_0.05_ in SC-Trp medium containing 2% gal and were incubated at 30°C.  Growth of the cultures was followed by their absorbance at OD_600_.  The experiment was repeated twice in duplicate with similar results.  Data represent the mean with standard deviations indicated by error bars.

*sgs1 top3* double mutant cells transformed with YEp112SpGAL or YEp112SpGAL-WRN were streaked onto plates containing SC minus Trp media with 2% gal.  As shown in Figure 2A, the WRN transformed *sgs1 top3* strain grew significantly slowly compared to the vector transformed *sgs1 top3* strain.  *sgs1 top3* transformed with the YEp112SpGAL-*SGS1* plasmid was included as a positive control (Figure 2A), demonstrating that wild-type Sgs1 expressed in the *sgs1 top3* mutant was able to genetically complement the growth phenotype.  The transformed *sgs1 top3* strains grew similarly in the absence of gal as shown for 2 days (Figure 2A) or earlier time points (data not shown).  Expression of WRN had no effect on the growth of parental wild-type (W3031A) or *sgs1* strains (Figure 2B), indicating that the effect of WRN on cell growth was specific to that observed in the *sgs1 top3* mutant background.

To further evaluate the effect of WRN expression on growth of the *sgs1 top3* double mutant, liquid culture experiments were performed.  After an initial lag phase for all cultures, *sgs1 top3*/WRN exhibited decreased growth compared to *sgs1 top3/*vector, evident at 12, 16, and 20 hr (Figure 2C). The decrease in cell growth for the *sgs1 top3/WRN* cells was not quite as great as that observed for *sgs1 top3/SGS1*, suggesting a partial restoration of the *top3*-associated growth phenotype by WRN.

### WRN expression alters the cell cycle distribution of *sgs1 top3*

**Figure 3.**
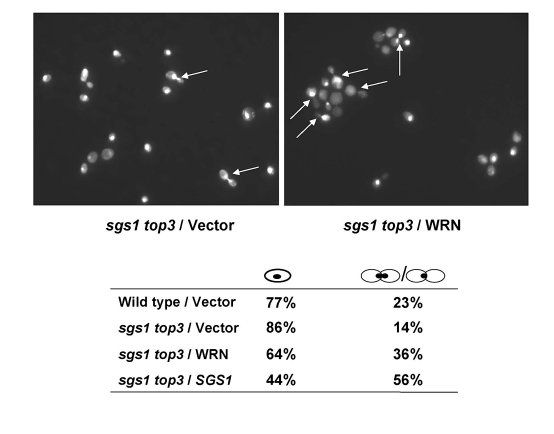
WRN expression induces S/G2 arrest in *sgs1 top3* cells. Logarithmically growing cultures of *sgs1 top3 *strain transformed with YEp112SpGAL, YEp112SpGAL-WRN, or YEp112SpGAL-SGS1, and the vector-transformed wild-type parental strain were induced at 2% gal concentration for 6 h.  Cultures were harvested, processed for DAPI staining as described in "Materials and Methods" and were observed using Axiovert 200 M microscope(Zeiss; 100x lens).  Shown is the DAPI staining of the *sgs1 top3* transformed with YEp112SpGAL (upper left) and with YEp112SpGAL-WRN (upper right).  Arrows show cells with undivided nuclei. Distribution of the cells in G1 (single cells) and S/G2 (budded cells) is shown in lower panel.

It was previously shown that *top3* mutant strains are delayed in the late S/G2 phase of the cell cycle [5], a characteristic that may account for their slow growth.  Mutation of the *SGS1* gene in the *top3* background suppresses the delay in the S/G2 phase of the cell cycle; consequently, the characteristic dumbbell shaped morphology of the *top3* mutant yeast cells that have not completed cell division is suppressed by the *sgs1* mutation. We examined if WRN expression could restore the delay in the S/G2 phase of the cell cycle in *sgs1 top3*.  An elevated population of large budded cells with undivided nuclei was observed for the *sgs1 top3* mutant cells expressing WRN (Figure 3).  The percentage of *sgs1 top3*/*WRN* cells with dumbbell morphology was approximately 2.6-fold greater than that of*sgs1 top3/vector* cells, suggesting that WRN expression in the *sgs1 top3* mutant restored the characteristic S/G2 delay of *top3* and this may contribute to the suppression of growth by WRN in the *sgs1 top3* cells.

### WRN ATPase/ helicase but not exonuclease activity is required to restore the *top3* slow growth phenotype

**Figure 4.**
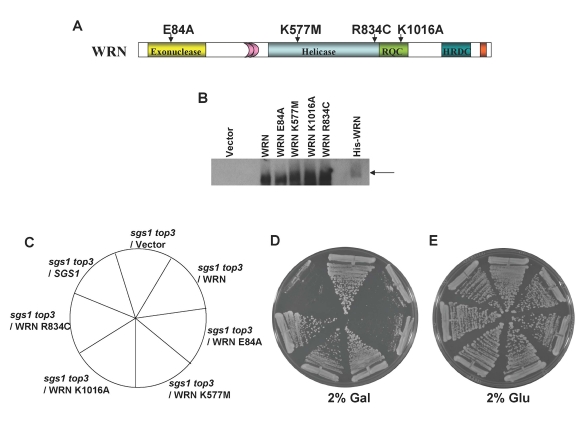
WRN ATPase/helicase, but not exonuclease activity, is required to restore the slow growth phenotype of *top3* in *sgs1 top3* background. *Panel **A***, WRN protein with conserved domains and positions of site-directed mutations.  *Panel* ***B***, Expression of WRN and WRN variants in transformed sgs1 top3 was induced at 2% gal concentration and cells were harvested after 6 h.  Equal amount of total cell lysate was loaded on to 8-16% polyacrylamide SDS gels, followed by Western blotting using anti-WRN antibody. *sgs1 top3* strain transformed with ATPase/helicase-dead (YEp195SpGAL-WRN K577M), exonuclease-dead (YEp195SpGAL-WRN E84A), RQC mutant (YEp195SpGAL-WRN K1016A), or polymorphic mutant (YEp195SpGAL-WRN R834C) was streaked on SC-Trp plates containing either 2% glu *(Panel **E**)* or 2% gal *(Panel **D**)*.  Plates were incubated at 30°C for 2 days and then photographed.  Composition of the plates was as in *Panel **C**.*

To assess the functional requirements of WRN to restore the slow growth phenotype in the *sgs1 top3* background, we assessed the importance of WRN catalytic activities (helicase, exonuclease) for slow growth phenotype restoration. To do this, we constructed WRN expression plasmids containing missense point mutations in the active site of the conserved exonuclease domain (E84A) or the ATPase/helicase domain (K577M) that were previously shown to inactivate the respective catalytic activities of the purified recombinant proteins [34,35] (Figure 4A).  Plasmids encoding the *WRN* mutant alleles were transformed into the*sgs1 top3* cells and expression of the  mutant WRN proteins was  confirmed by  Western blot analyses (Figure 4B).  The effects of mutant WRN protein expression on growth were assessed by streaking the transformed yeast strains onto SC plates media with 2% gal but lacking Trp.  Streak analysis revealed that the WRN-E84A exonuclease dead mutant restored the* top3*-associated slow growth phenotype in *sgs1 top3* comparable to wild-type WRN, whereas the WRN-K577M ATPase/ helicase dead mutant failed to restore the slow growth in the*sgs1 top3* as shown by its similar level of growth to that of the *sgs1 top3*/vector cells (Figure 4D).  However, the *sgs1 top3*/WRN-E84A and *sgs1 top3*/WRN-K577M cells grew equally well in the absence of gal induction (Figure 4E).  From these results, we conclude that WRN helicase but not exonuclease activity was required for restoration of slow growth phenotype characteristic of *top3* in the *sgs1 top3* background.

### Genetic analysis of a naturally occurring WRN missense polymorphism or engineered RQC domain WRN missense mutant in the *sgs1 top3 *background

In addition to the helicase domain, many RecQ helicases share another conserved sequence C-terminal to the ATPase/helicase core domain designated the RecQ C-terminal (RQC) region that has been implicated in DNA binding and protein interactions [4].  To address the potential importance of the WRN RQC domain for its biological function, we examined the ability of a specific WRN RQC missense mutant, WRN-K1016A (Figure 4A), to restore the slow growth phenotype of *top3* in *sgs1 top3*. The WRN-K1016A mutant was previously characterized and shown to have significantly reduced DNA binding and helicase activity compared to the wild-type recombinant WRN protein [36].  *sgs1 top3* expressing WRN-K1016A (Figure 4B) grew similarly to the *sgs1 top3*/vector cells (Figure 4D), indicating that expression of the WRN-K1016A mutant failed to restore the slow growth phenotype characteristic of the *top3* mutant.

Although no missense mutations have been identified that are genetically linked to Werner syndrome, a number of WRN polymorphic missense variants exist whose biological significance is not well understood.  The lack of genetic data on WRN polymorphisms suggested to us that the yeast-based WRN system might be useful in their analysis.  The WRN-834C polymorphism residing in the core helicase domain wasidentified in DNA from the Polymorphism Discovery Resource database (egp.gs.washington.edu) (Figure 4A).  Previously, it was reported by the Loeb lab that the purified recombinant WRN-R834C protein has dramatically reduced WRN ATPase, helicase, and helicase-coupled exonuclease activity [37].  To assess the biological effect of the WRN R834C polymorphic change, *sgs1 top3*/WRN-R834C was streaked onto a SC plate lacking Trp and containing 2% gal.  Expression of WRN-R834C (Figure 4B) demonstrated comparable growth to that of *sgs1 top3*/vector (Figure 4D).  These results demonstrate that the WRN-R834C polymorphism, similar to the K577M and K1016A mutations that interfere with WRN helicase activity, interfere with WRN function in the *sgs1top3* background.

**Figure 5.**
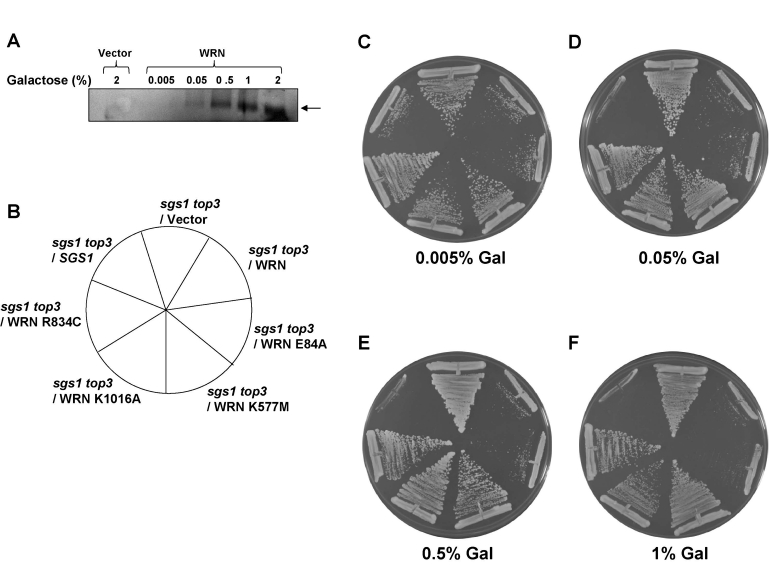
WRN mediated restoration of slow growth phenotype in *sgs1 top3* background is independent of its expression level. WRN expression in the transformed *sgs1 top3* cells was induced with the indicated gal concentrations and cells were harvested 6 h after induction.  As a control,* sgs1 top3*/YEp112SpGAL was included.  Equal amounts of total cell lysate were loaded on  8-16%  polyacrylamide SDS  gels followed by Western blot detection using  anti-WRN antibody  as  shown in *Panel* **A**. sgs1 top3 strain transformed with YEp112SpGAL, YEp112SpGAL-WRN, exonuclease-dead (YEp195SpGAL-WRN E84A), ATPase/helicase-dead (YEp195SpGAL-WRN K577M), RQC mutant (YEp195SpGAL-WRN K1016A), polymorphic mutant(YEp195SpGAL-WRN R834C) and YEp112SpGAL-SGS1 was streaked on SC-Trp plate containing gal at varying concentrations asindicated. Plates were incubated at 30°C for 2 days and then photographed. Panels **C-F** show the effect of WRN expression onthe growth rate of sgs1 top3 transformed strains at different gal concentrations. Composition of the plates was as in *Panel* **B**.

Previous studieshave shown that gene expression levels from the GAL*1/10* promotorcan be regulated by altering the concentration of gal in thegrowth medium.  We therefore examined the level of WRN protein expression in cells grown at lower gal concentrations (0-2%) in the presence of 2% raffinose (raf).  Western blot analyses demonstrated that WRN expression was dependent on the concentration of gal in the media (Figure 5A). Quantitative Western blot analyses using purified recombinant WRN protein as standard indicated that 2.3 x 10^4^WRN molecules/cell were present at 2% gal.  As WRN expression was regulated by the level of gal, we wanted to compare the ability of WRN and its associated variants to restore the slow growth phenotype of *top3* in *sgs1 top3* background at lower levels  of protein expression.  WRN restored the slow growth phenotype of *sgs1 top3* throughout the gal concentration range, including the lower gal concentrations (Figure 5C-F).  Similarly, WRN-E84A mutant restored the slow growth *top3* phenotype, whereas the ATPase/ helicase dead WRN-K577M mutant failed to do so (Figure 5C-F).  Likewise, neither WRN-R834C nor WRN-K1016A affected the growth of the *sgs1 top3* mutant (Figure 5C-F). No effect on the growth rate of wild-type parental strain (W3031B) as well as *sgs1* strain was observed with WRN or its associated variants (Supp. Data Figures 2 and 3).  These results demonstrate that wild-type WRN or the WRN exonuclease dead proteins are similarly able to restore the *top3* growth phenotype in the *sgs1 top3 *background, whereas the WRN mutant proteins that have defective ATPase/helicase activity did not affect the growth of the *sgs1 top3* mutant at either low or high levels of gal-induced protein expression.

### Effect of WRN expression on the MMS and HU sensitivity of the *sgs1 top3* mutant

**Figure 6.**
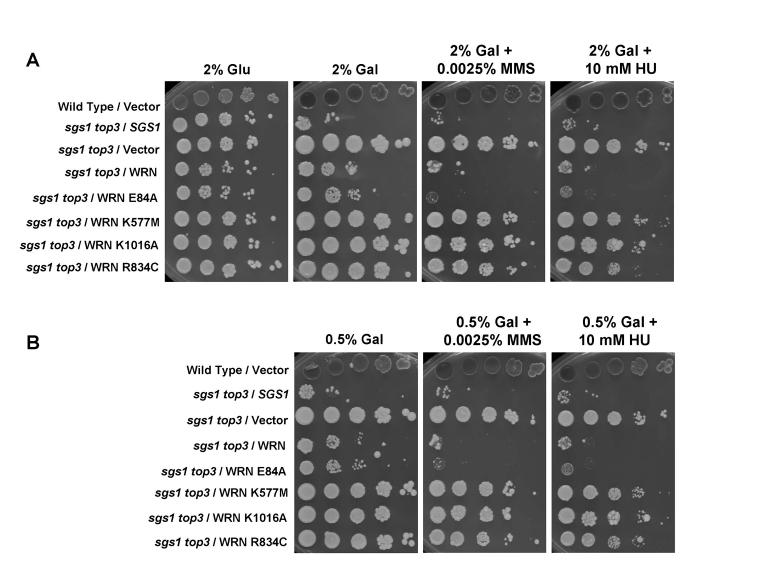
Effect of WRN expression on the MMS and HU sensitivity of *sgs1 top3* strain. Logarithmically growing cultures of *sgs1 top3 *strain transformed with YEp112SpGAL, YEp112SpGAL-WRN, exonuclease-dead (YEp195SpGAL-WRN E84A), ATPase/helicase-dead (YEp195SpGAL-WRN K577M), RQC mutant (YEp195SpGAL-WRN K1016A), polymorphic mutant (YEp195SpGAL-WRN R834C), YEp112SpGAL-*SGS1 *and vector transformed wild type parental strains were spotted in a ten-fold serial dilutions onto SC-Trp plates containing glu or gal and either MMS or HU at the indicated concentrations.  Plates were incubated at 30°C for 2 days (control plates) and 4 days (MMS and HU plates) and then photographed.

Previously, it was reported that the *sgs1 top3* double mutant is less sensitive to MMS or HU than the *top3 *single mutant [6].  Since WRN affected cell growth of *sgs1 top3*, we next examined its effect on drug sensitivity.  As shown in Figure 6, *sgs1 top3*/WRN displayed sensitivity to both MMS and HU that was comparable to *sgs1 top3/SGS1, *whereas *sgs1 top3*/vector was more resistant to either drug.  Genetic analysis of the WRN variants in the *sgs1 top3* background revealed that strains transformed with WRN-K577M,   WRN-K1016A, or  WRN-R834C  displayed sensitivity comparable to that of vector, whereas the *sgs1 top3*/WRN-E84A strain showed HU and MMS resistance similar to *sgs1 top*/WRN and s*gs1 top3/SGS1 *(Figure 6).  These results demonstrate that in addition to its effect on growth in the *sgs1 top3 *background, WRN also affects sensitivity of the *sgs1 top3* mutant to HU or MMS.  Furthermore, WRN ATPase/helicase but not exonuclease activity is genetically required for its effect on HU or MMS sensitivity in the *sgs1 top3* background.  Expression of WRN in the wild-type parent strain had no effect on the strain's sensitivity to the tested concentration of HU or MMS (Supp. Data Figure 4), indicating that the effect of WRN expression was dependent on the *sgs1 top3* genetic background.

## Discussion

From our studies, we conclude that WRN genetically interacts with Top3 through its ability to restore the* top3* slow growth phenotype in the *sgs1 top3* background.  WRN helicase activity is required for genetic restoration of slow growth, similar to previous studies which demonstrate that *sgs1 *helicase defective alleles fail to suppress *top3* slow growth [38,39]. Although WRN suppressed the growth in the *sgs1 top3 *double mutant, WRN did not rescue the sensitivity of *sgs1* to the DNA damaging agent MMS or the replication inhibitor HU.  Thus, WRN cannot directly replace the Sgs1 helicase in the cellular response to replicational stress.  However, in a pathway defined by genetic background, human WRN expressed in yeast can clearly exert a phenotype.  The ability of WRN to affect the growth phenotype in the *sgs1 top3* background is supported by our observation that the morphological appearance of large budded cells with undivided nuclei characteristic of S/G2 arrest is restored in the WRN-transformed *sgs1 top3* mutant.  This work is the first demonstration that WRN can function in a genetic pathway that affects *top3-*related phenotypes, and this role is dependent on WRN helicase but not exonuclease activity.

Previous studies demonstrated that expression of WRN or BLM in *sgs1 *does not affect growth rate, but can partially suppress illegitimate recombination or homologous recombination [40].  However, a distinction in the biological functions between the two human RecQ helicases was suggested based on the observation that expression of BLM, but not WRN, can restore the resistance of the *sgs1* mutant to the replication inhibitor HU [40] and extend the shortened lifespan caused by the *sgs1* mutation [41].  It was also reported that human BLM, but not WRN, can suppress the growth in the *sgs1 top3* double mutant [40], suggesting that yeast Sgs1 helicase has functions similar to those of human BLM helicase. Although we also observed that WRN expression failed to correct the HU sensitivity of the *sgs1* mutant, we found that WRN expression can restore the *top3* slow growth phenotype in the *sgs1 top3* background.  The difference between our study and the earlier one may reflect differences in WRN protein expression (since the earlier study did not report a quantitative level of WRN protein), strains, or yeast culture conditions.  Our quantitative Western blot analyses demonstrate that WRN expressed in yeast at levels comparable to that previously reported in several human cell lines was sufficient to restore growth in the *sgs1 top3* mutant as detected by plate streak studies or liquid culture growth analysis. From these results, we conclude that WRN can function in the *sgs1 top3 *background, indicating some genetic overlap between the WRN and Sgs1 pathways.  By inference, WRN may also have overlapping genetic functions with BLM.  Although WRN was not observed *in vitro* to substitute in the BLM-Top3α complex double HJ dissolution reaction (see Introduction), it is plausible that WRN interacts with Top3α in a related protein complex with additional factors and can perform a function important for genomic stability.  The results from our *sgs1 top3 *WRN complementation studies in yeast prompt further investigation of the possibility that WRN functionally interacts with Top3α in human cells during cellular DNA replication or recombination. Conceivably, in a BLM-impaired condition, WRN may partially substitute for BLM through its protein partnership with a topoisomerase.

In a previous study, we found that human WRN expressed in a yeast *dna2-1 *mutant background can rescue the associated replication and repair phenotypes; however, a non-catalytic C-terminal domain of WRN was sufficient for genetic rescue, demonstrating that WRN helicase activity is not required to rescue the mutant cellular phenotypes associated with the *dna2-1 *mutant [31].  Based on genetic and biochemical results, the explanation for this finding was that WRN rescues the *dna2-1 *mutant by interacting with endogenous yeast Flap Endonuclease-1 (FEN-1) and stimulating its nuclease activity, thereby bypassing the requirement for wild-type DNA2 nuclease to process DNA replication and repair intermediates [31,42].  In contrast, we show in the current study that WRN helicase activity is required to suppress growth in the* sgs1 top3 *mutant.  The distinct genetic requirements for WRN to rescue different mutant backgrounds suggest that WRN can operate in separate pathways using different catalytic or protein interaction domains.  We propose that WRN is a modular pleiotropic protein with unique catalytic or protein interaction domains that are necessary to fulfill its functions in different biological settings dictated by mutant genetic background.

In addition to the yeast studies which have examined the genetic interactions between *sgs1* and *top3*, the potential functional overlap of Sgs1 with other topo-isomerases has been investigated.  For example, *sgs1 top1 *mutants are severely growth impaired, suggesting that the synergistic defect can be attributed to the two genes operating in separate but overlapping pathways. This is distinct from the epistatic relationship of *sgs1* and *top3*. Based on their genetic findings, Weinstein and Rothstein proposed that a subset of DNA structures which arise at stalled or collapsed replication forks normally processed by the Sgs1-Top3 pathway can be alternatively channeled into a Top1-dependent pathway in the absence of active Sgs1 helicase activity [39].  It is conceivable that WRN, which interacts physically and functionally with human TOP1 [43], may collaborate with Top1 (or another topoisomerase) in yeast to generate a viable but poorly growing cell in a pathway parallel to Sgs1.  Alternatively, WRN may directly substitute for Sgs1 only in a specialized situation when Top3 is absent.  It is conceivable that the poor growth of the *top3* mutant is attributed to unresolved recombinogenic DNA intermediates created by WRN helicase activity.  This last idea underscores a growing notion in the field that genetic recombination must be properly regulated; otherwise, deleterious recombination events acting on aberrant DNA structures prevail [44].  Thus, RecQ helicase-mediated recombination pathways are like a double-edged sword. In the appropriate genetic background, these pathways secure a normal growth rate and genomic stability; however, in certain mutant backgrounds (e.g., *top3*), DNA unwinding by a RecQ helicase is counter-productive to cell growth and genome homeostasis.

## Materials and methods

Plasmid DNA constructs. Site-directed mutations of WRN (E84A, K577M, R834C, and K1016A) in the plasmid YEp195SpGAL-WRN [31] were constructed using mutagenic primers (Supplementary Table 1) and a standard protocol from Quickchange II XL site-directed mutagenesis kit (Stratagene) by Lofstrand labs (Gaithersburg, MD). DNA fragments encoding WRN and its associated variants were gel purified from the respective YEp195SpGAL-WRN constructs after double digestion with *Sal*I and *Mlu*I.  Gel purified fragments were then cloned into the *Sal*I-*Mlu*I sites of vector YEp112SpGAL [45], a 2 μm multi-copyplasmid containing a *TRP1* selectable marker to construct YEp112SpGAL-WRNor WRN variants under the control ofa gal-inducible promotor.  The *SGS1* expression plasmid pSGS1f2 was kindly provided by Dr. Brad Johnson (University of Pennsylvania School of Medicine, Philadelphia, Pennsylvania).  *SGS1* was PCR amplified using  5'-ACGCGTCGACATGGTGACGAA GC-3' and 5'-GTCTCCTTCACTACGCGTCGAAT-3' as the forward and the reverse primers, respectively, using pSGS1f2 as a template. PCR was carried out with PCR super mix HiFi (Invitrogen) for 30 cycles (denaturation at 94°C for 30 s per cycle, annealing at 57°C for 50 s per cycle, and elongation at 72°C for 5 min per cycle).  The amplified product was cloned into the *Sal*I-*Mlu*I sites of vector YEp112SpGAL.

Yeast media and strains. Strains with wild-type *SGS1 TOP3 * (WT; W303-1A, genotype, *MAT**a** ade2-1 canl- 100 his3-11,15 leu2-3,112 trpl-l ura3-1*) [5], a *sgs1 *mutant (W1292-3C; genotype *MAT***a ***SUP4-***o***::URA3 sgs1-25 ade2-1 can1-100 his3-11,15 leu2-3,112 trp1-1 ura3-1 rad5-535*) and a *sgs1 top3* mutant ( W1058-11C, genotype, *MAT***a ***SUP4-***o***::URA3 sgs1-25 top3-2::HIS3 ade2-1 can1-100 his3-11,15 leu2-3,112 trp1-1 ura3-1 rad5-535)* have been characterized [6] and were kindly provided by Dr. Rodney Rothstein (Columbia University).  Yeast cultures were grown using standard protocol and transformations were performed using a Lithium Acetate-based protocol [46]. Transformed yeast strains were grown in SC media minus Trp and containing either glucose (glu) or gal as required.

Genetic analysis of hydroxyurea or methylmethane-sulfonate sensitivity in WRN transformed yeast strains. To determine the effect of WRN expression on the MMS and HU sensitivity of *sgs1* or *sgs1 top3* strains, strains transformed with YEp112SpGAL or YEp112SpGAL-WRN were grown in SC raf minus Trp at 30^o^C. Cultures were reinoculated in SC raf minus Trp and grown at 30°C to early log phase (OD_600_ of ~0.6 to 0.8).  Ten-fold serial dilutions of these strains were spotted onto SC gal minus Trp plates containing the indicated concentrations of HU or MMS.  Plates were incubated at 30°C.  As a control, wild-type parental strain (W303-1A) transformed with YEp112SpGAL or YEp112SpGAL-WRN, or *sgs1 top3*/ YEp112SpGAL-*SGS1* was treated as described above.

Genetic analysis of the slow growth phenotype in WRN transformed *sgs1 top3* strain. To examine the effect of WRN expression on the growth of wild-type parental (W303-1A), *sgs1*, or *sgs1 top3* strains, the corresponding strains transformed with YEp112SpGAL or YEp112SpGAL-WRN were streaked onto SC minus Trp plates containing either glu or gal as indicated.  As a control, *sgs1 top3*/YEp112SpGAL-*SGS1* was included. Plates were incubated at 30°C.  To determine the ability of WRN to affect growth rate of *sgs1 top3* strain at lower WRN protein expression levels, the *sgs1 top3* strain transformed with YEp112SpGAL, YEp112SpGAL-WRN, or YEp112SpGAL-*SGS1* was streaked onto SC minus Trp plates containing varying concentrations of gal as indicated.

To assess the functional requirements of WRN to restore the slow growth phenotype in the *sgs1 top3* background, *sgs1 top3* transformed with YEp112SpGAL-WRN (E84A, K577M, R834C, and K1016A) were streaked onto SC minus Trp plates containing either glu or gal as indicated. For controls, *sgs1 top3* strain transformed with YEp112SpGAL,  YEp112SpGAL-WRN or  YEp112Sp GAL-*SGS1* was included.

To evaluate the growth of transformed *sgs1 top3* strains in liquid culture, yeast cells were grown in SC raf minus Trp at 30^o^C. Cultures were reinoculated in SC raf minus Trp and grown at 30°C to early log phase (*A *_600_ of ~0.5).  Cultures were then reinnoculated at OD_0.05_ in media containing 2% gal and their growth was followed by measuring absorbance at the indicated time intervals.

Cell cycle distribution of WRN transformed *sgs1 top3* strain. Logarithmically growing cultures of *sgs1 top3* transformed with YEp112SpGAL, YEp112SpGAL-WRN,  or YEp112SpGAL-SGS1, and the vector-transformed wild-type parental strain were induced at 2% gal concentration for 6 h.  For DAPI (4', 6-diamidino-2-phenylindole) staining, 70% ethanolfixed cells were washed with phosphate-buffered saline (PBS), stained and mounted in Vectashield mounting medium with DAPI (1 μg/ml) (Vector,USA).  Cells were examined with an Axiovert 200 M microscope(Zeiss; 100x lens) and composite differential interference contrast(DIC) and fluorescence images were analyzed using AxioVision,version 3.0 program (Zeiss).

Western blot analyses. Transformed *sgs1* or *sgs1 top3 *strains were grown in SC raf minus Trp at 30^o^C to an OD_600_ of 0.5.  Cultures were then induced at the indicated gal concentration for 6 h. Cells (3 ml) were collected by centrifugation, washed with PBS, lysed in alkaline lysis buffer [50 mM NaOH, pH 10.5, 2 mM EDTA, 1 mM phenylmethylsulfonyl fluoride (PMSF), 2% SDS, 10% glycerol, 5% 2-mercaptoethanol and protease inhibitors (Roche Molecular Biochemicals)], boiled for 5 min, clarified by centrifugation, and neutralized with 1 M HCl. Proteins from equivalent amounts of cell lysate were resolved on 8-16% polyacrylamide SDS gels.  Expression of WRN or WRN mutant proteins was determined by Western blot using a WRN mouse monoclonal antibody directed against an epitope in a purified C-terminal fragment of WRN [47] (1:1000, Spring Valley Labs).  For quantitative Western blot analysis, increasing concentrations of purified recombinant His-tagged full-length WRN protein were included on gels with yeast lysate samples.

## Supplementary data

Supplementary Figure 1 WRN fails to rescue the MMS and HU sensitivity of *sgs1*. Cultures of wild-type parental strain (W303-1A) or *sgs1* strain transformed with YEp112SpGAL or YEp112SpGAL-WRN were grown to early log phase (*OD *_600_ of ~0.6 to 0.8). Ten-fold serial dilutions of these cultures were spotted onto SC-Trp plates containing 0.5% gal and either MMS or HU at the indicated concentrations. Plates were incubated at 30°C for 3 days (control plates) and 5 days (MMS or HU plates) and then photographed.

Supplementary Figure 2 WRN expression has no effect on the growth rate of wild type parental strain W303-1A. Wild type parental strain W303-1A transformed with YEp112SpGAL, YEp112SpGAL-WRN, exonuclease-dead (YEp195SpGAL-WRN E84A), ATPase/helicase-dead (YEp195SpGAL-WRN K577M), RQC mutant (YEp195SpGAL-WRN K1016A) and polymorphic mutant (YEp195SpGAL-WRN R834C) was streaked on SC-Trp plate containg either 2% glu (*Panel **B***) or galactose at varying concentrations as indicated (*Panel **C-G***). Plates were incubated at 30°C for 2 days and then photographed. Composition of the plates was as in *Panel **A**.*

Supplementary Figure 3 WRN expression has no effect on the growth rate of *sgs1* strain. *sgs1* strain transformed with YEp112SpGAL, YEp112SpGAL-WRN, exonuclease-dead (YEp195SpGAL-WRN E84A), ATPase/helicase-dead (YEp195SpGAL-WRN K577M), RQC mutant (YEp195SpGAL-WRN K1016A) and polymorphic mutant (YEp195SpGAL-WRN R834C) was streaked on SC-Trp plate containing either 2% glu (*Panel **B***) or gal at varying concentrations as indicated (*Panel **C-G***).  Plates were incubated at 30°C for 4 days and then photographed.  Composition of the plates was as in *Panel **A**.*

Supplementary Figure 4 Effect of WRN expression on the MMS and HU sensitivity of wild-type parental strain W303-1A. Logarithmically growing cultures wild type parental strain transformed with YEp112SpGAL or YEp112SpGAL-WRN was spotted in a ten-fold serial dilutions onto SC-Trp plates containing glu or gal and either MMS or HU at the indicated concentrations.  Plates were incubated at 30°C for 2 days.

Supplementary Table 1 Oligonucleotides used for WRN site-directed mutagenesis.
