# Supplementary figures and images for: WRN helicase defective in the premature aging disorder Werner syndrome genetically interacts with topoisomerase 3 and restores the top3 slow growth phenotype of sgs1 top3
Source: Aging (Albany NY). 2009 Feb 5;1(2):219–33. doi: 10.18632/aging.100020 (PMC2806000; doi:10.18632/aging.100020)

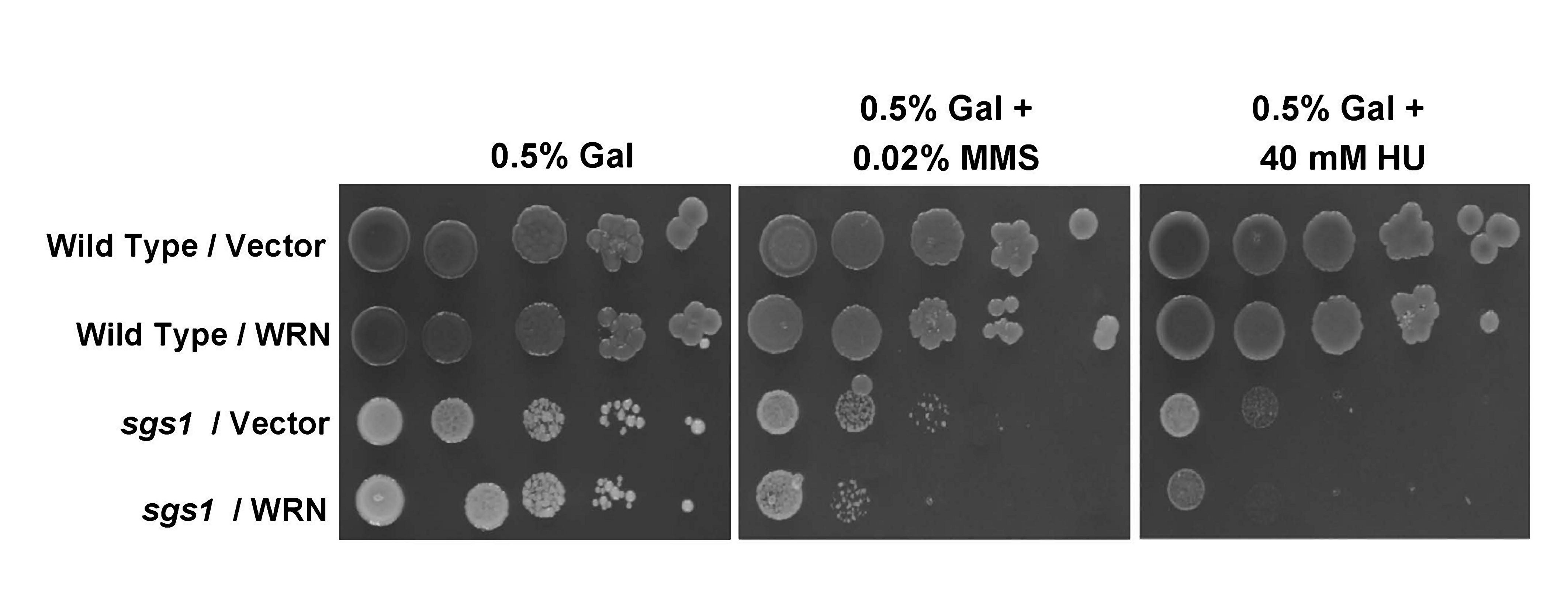

Supplement: Supplementary Figure 1 — Cultures of wild-type parental strain (W303-1A) or sgs1 strain transformed with YEp112SpGAL or YEp112SpGAL-WRN were grown to early log phase (OD 600 of ~0.6 to 0.8). Ten-fold serial dilutions of these cultures were spotted onto SC-Trp plates containing 0.5% gal and either MMS or HU at the indicated concentrations. Plates were incubated at 30°C for 3 days (control plates) and 5 days (MMS or HU plates) and then photographed. [file aging-01-219-s001.tif]

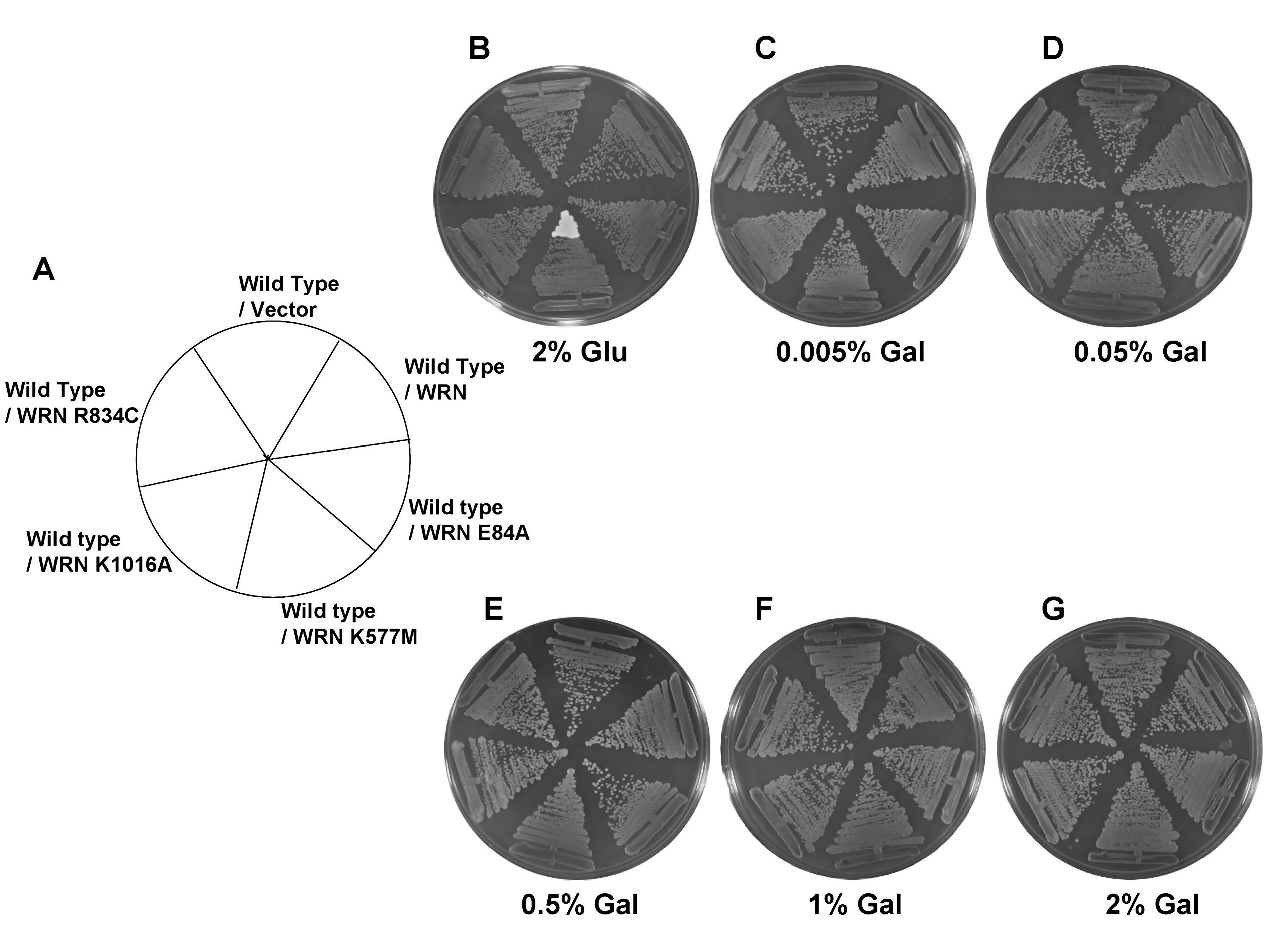

Supplement: Supplementary Figure 2 — Wild type parental strain W303-1A transformed with YEp112SpGAL, YEp112SpGAL-WRN, exonuclease-dead (YEp195SpGAL-WRN E84A), ATPase/helicase-dead (YEp195SpGAL-WRN K577M), RQC mutant (YEp195SpGAL-WRN K1016A) and polymorphic mutant (YEp195SpGAL-WRN R834C) was streaked on SC-Trp plate containg either 2% glu (Panel B) or galactose at varying concentrations as indicated (Panel C-G). Plates were incubated at 30°C for 2 days and then photographed. Composition of the plates was as in Panel A. [file aging-01-219-s002.tif]

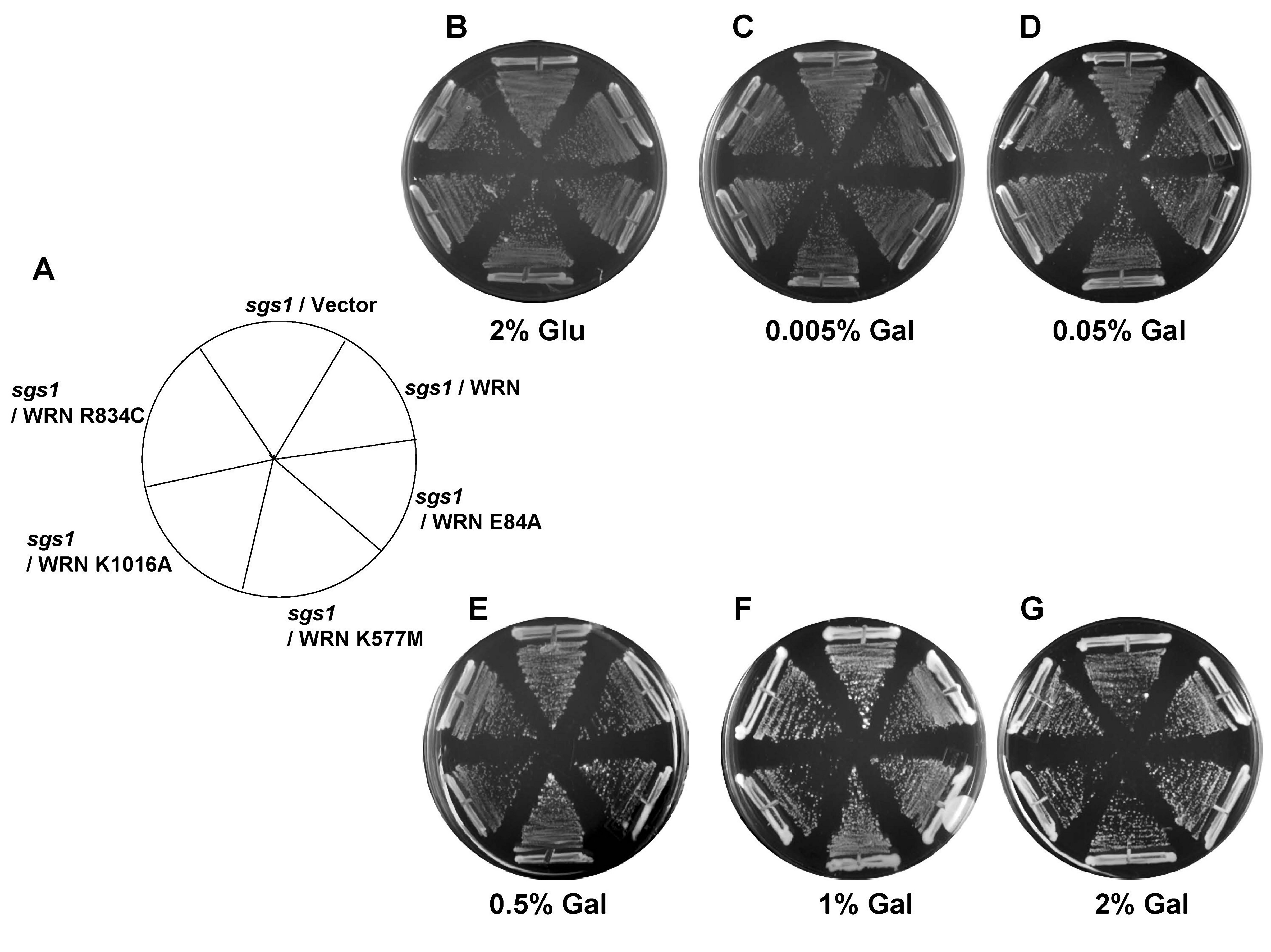

Supplement: Supplementary Figure 3 — sgs1 strain transformed with YEp112SpGAL, YEp112SpGAL-WRN, exonuclease-dead (YEp195SpGAL-WRN E84A), ATPase/helicase-dead (YEp195SpGAL-WRN K577M), RQC mutant (YEp195SpGAL-WRN K1016A) and polymorphic mutant (YEp195SpGAL-WRN R834C) was streaked on SC-Trp plate containing either 2% glu (Panel B) or gal at varying concentrations as indicated (Panel C-G). Plates were incubated at 30°C for 4 days and then photographed. Composition of the plates was as in Panel A. [file aging-01-219-s003.tif]

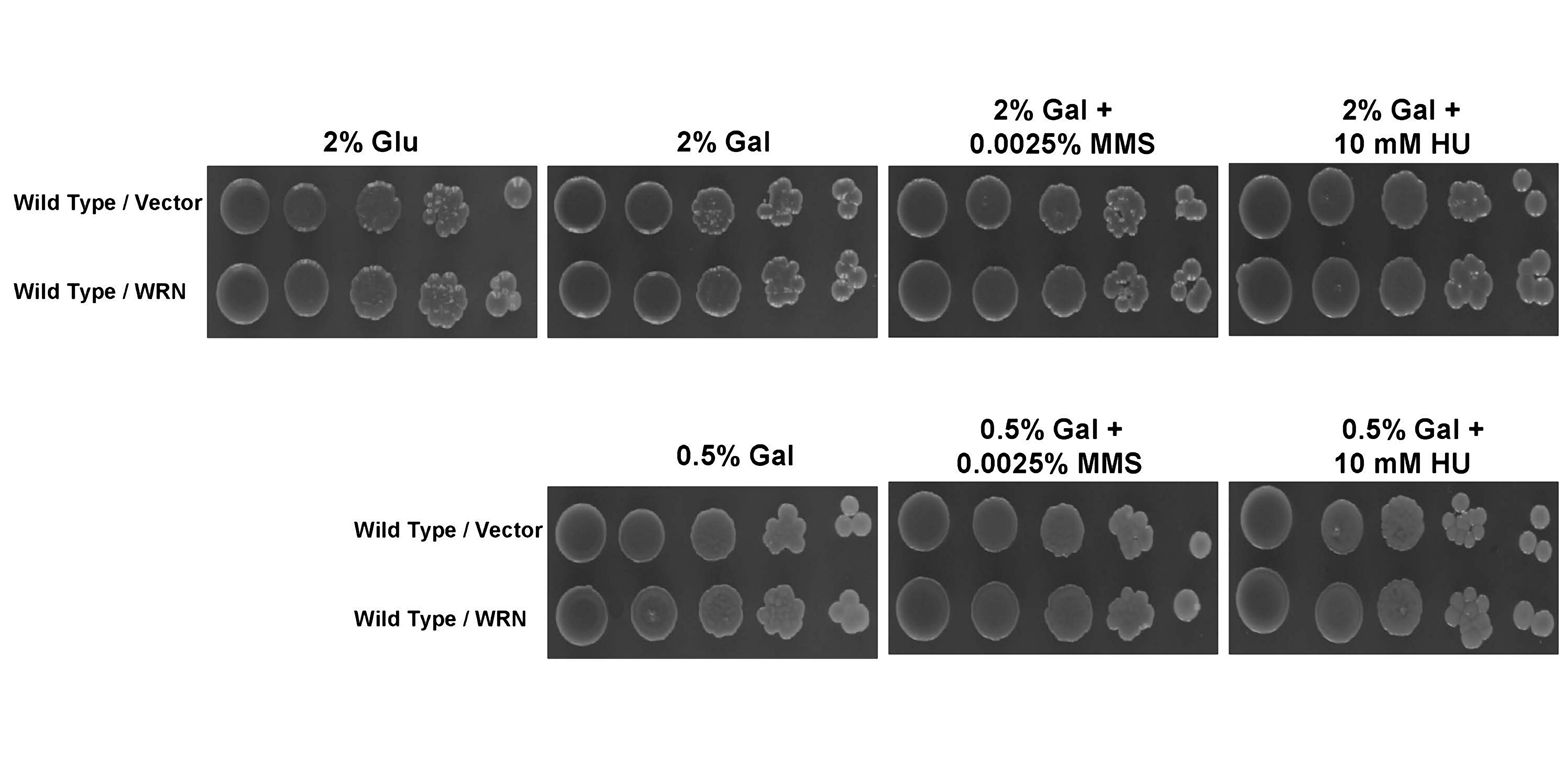

Supplement: Supplementary Figure 4 — Logarithmically growing cultures wild type parental strain transformed with YEp112SpGAL or YEp112SpGAL-WRN was spotted in a ten-fold serial dilutions onto SC-Trp plates containing glu or gal and either MMS or HU at the indicated concentrations. Plates were incubated at 30°C for 2 days. [file aging-01-219-s004.tif]
