# Supplementary material for: WRN helicase defective in the premature aging disorder Werner syndrome genetically interacts with topoisomerase 3 and restores the top3 slow growth phenotype of sgs1 top3
Source: Aging (Albany NY). 2009 Feb 5;1(2):219–33. doi: 10.18632/aging.100020 (PMC2806000; doi:10.18632/aging.100020)
Supplement: Supplementary file 2 [file aging-01-219-s001.doc]

| Residue Mutated | Primers used |
|---|---|
| E84A | 5'-GGG ATT TGA CAT GG**C** GTG GCC ACC ATT ATA C-3'<br>3'- CCC TAA ACT GTA CC**G** CAC CGG TGG TAA TAT G-5' |
| K577M | 5'-GGC AAC TGG ATA TGG AA**T** GAG TTT GTG CTT CC-3'<br>3'- CCG TTG ACC TAT ACC TT**A** CTC AAA CAC GAA GG-5' |
| R834C | 5'-AAT AAA GCT GAC ATT **T**GC CAA GTC ATT CAT TAC -3'<br>3'-TTA TTT CGA CTG TAA **A**CG GTT CAG TAA GTA ATG-5' |
| K1016A | 5'-CAG AGA GTT GGT GG**G** **C**GG CTT TTT CCC GTC AGC -3'<br>3'-GTC TCT CAA CCA CC**C** **G**CC GAA AAA GGG CAG TCG -5' |
